# Dose prediction of organs at risk in patients with cervical cancer receiving brachytherapy using needle insertion based on a neural network method

**DOI:** 10.1186/s12885-023-10875-6

**Published:** 2023-04-28

**Authors:** Huai-wen Zhang, Xiao-ming Zhong, Zhen-hua Zhang, Hao-wen Pang

**Affiliations:** 1 Department of Radiotherapy, Jiangxi Cancer Hospital, The Second Affiliated Hospital of Nanchang Medical College, Jiangxi Clinical Research Center for Cancer, Nanchang, 330029 China; 2 Department of Oncology, The third people’s hospital of Jingdezhen, Jingdezhen, 333000 China; 3 Department of Oncology, The Affiliated Hospital of Southwest Medical University, Luzhou, 646000 China

**Keywords:** Brachytherapy, Needle insertion, Neural network, Dose prediction

## Abstract

**Objective:**

A neural network method was employed to establish a dose prediction model for organs at risk (OAR) in patients with cervical cancer receiving brachytherapy using needle insertion.

**Methods:**

A total of 218 CT-based needle-insertion brachytherapy fraction plans for loco-regionally advanced cervical cancer treatment were analyzed in 59 patients. The sub-organ of OAR was automatically generated by self-written MATLAB, and the volume of the sub-organ was read. Correlations between D2cm^3^ of each OAR and volume of each sub-organ—as well as high-risk clinical target volume for bladder, rectum, and sigmoid colon—were analyzed. We then established a neural network predictive model of D2cm^3^ of OAR using the matrix laboratory neural net. Of these plans, 70% were selected as the training set, 15% as the validation set, and 15% as the test set. The regression R value and mean squared error were subsequently used to evaluate the predictive model.

**Results:**

The D2cm^3^/D90 of each OAR was related to volume of each respective sub-organ. The R values for bladder, rectum, and sigmoid colon in the training set for the predictive model were 0.80513, 0.93421, and 0.95978, respectively. The ∆D2cm^3^/D90 for bladder, rectum, and sigmoid colon in all sets was 0.052 ± 0.044, 0.040 ± 0.032, and 0.041 ± 0.037, respectively. The MSE for bladder, rectum, and sigmoid colon in the training set for the predictive model was 4.779 × 10^−3^, 1.967 × 10^−3^ and 1.574 × 10^−3^, respectively.

**Conclusion:**

The neural network method based on a dose-prediction model of OAR in brachytherapy using needle insertion was simple and reliable. In addition, it only addressed volumes of sub-organs to predict the dose of OAR, which we believe is worthy of further promotion and application.

## Introduction

The incidence and mortality rates of cervical cancer are high among women worldwide [1], and concurrent radiotherapy and chemotherapy can significantly improve the local control rate in these patients [2]. Brachytherapy is a key technique used in the radical radiotherapy of cervical cancer, and possesses the distinct advantages of physical dosimetry, enabling the tumor to receive relatively high doses without causing serious complications to surrounding normal tissues [3]. However, locally advanced tumors are relatively large and frequently invade nearby cervical tissues. Conventional brachytherapy does not adequately enclose the target volume, often resulting in uncontrolled or recurrent tumors. In contrast, brachytherapy using needle insertion improves target coverage [4], thereby enhancing local control and overall patient survival [5, 6].

In designing a brachytherapy plan, the choice of constraint parameters is of utmost importance and directly influences the quality of the final plan. However, this information is unknown prior to designing the clinical brachytherapy plan. The plan designer usually refers to the optimization definition goal provided by the doctor, which is based on data from the general population data and radiation therapy group. The Radiation Therapy Oncology Group guidelines or statistically derived clinical norms serve as the target for OAR dose optimization [7]. In general, these reference objectives are universal. However, an optimal brachytherapy plan should be based upon each patient’s unique anatomic structures, and, therefore, methods that apply universal clinical norms cannot meet the needs of the individual patient.

In recent years, machine learning methods have been widely applied to external beam radiotherapy, and have shown promising results [8–11]. For the present study, we implemented the authorized Chinese invention patent method (patent number: ZL201610529290.8), which can predict the OAR dose distribution prior to the design of the brachytherapy plan. This can help the plan designer in evaluating the quality of the brachytherapy plan, determine whether the brachytherapy plan meets the requirements, provide standards for dosimetry verification and quality control, meet the specific needs of the individual patient, and provide a basis for the automation of tumor radiotherapy planning. To our knowledge, this work is the first to apply brachytherapy using needle insertion. This method can ensure dose distribution with high precision and thus improve the efficiency of brachytherapy.

## Materials and methods

### Patients

A total of 218 CT-based needle-insertion brachytherapy fraction plans were analyzed in 59 patients with loco-regionally advanced cervical cancer in the Department of Oncology of the Affiliated Hospital of Southwest Medical University. Of these plans, 70% were selected as the training set, 15% as the validation set, and 15% as the test set applying the matrix laboratory (MATLAB) neural net-fitting application.

All patients were scanned with contrast-enhanced magnetic resonance images (MRI, Achieva 3.0 T, Philips, Amsterdam, The Netherlands) and gynecological examinations were performed to identify tumor areas before external beam intensity-modulated radiation therapy (IMRT) and brachytherapy. All patients were administered diluted iohexol (10 mL in 1000 mL of water) as a gastrointestinal contrast medium before brachytherapy, and patients also received an enema and underwent bladder catheterization prior to treatment. Experienced physicians determined the location of the uterus and tumor characteristics based upon the results of the MRI and gynecological examinations, and the site of needle insertion.

### Target volume delineation and treatment planning

All patients underwent external beam IMRT at a prescribed dose of 45 Gy/25 F, which was increased to 60 Gy/25 F for patients with biopsy-positive lymph nodes. After external beam IMRT, ^192^Ir high-dose-rate brachytherapy was administered at a dose of 6 or 7 Gy/fraction, 1–2 fractions/week, for a total of 4 or 5 fractions. An appropriate intrauterine tube was inserted into the uterine cavity based on the depth and angle of the uterine cavity. Next, based on the tumor location, metal insertion needles were inserted into the tumor. Brachytherapy was performed on a ^192^Ir source (mHDR, Elekta, The Netherlands) using a Microelectron v2 afterloader (Elekta, The Netherlands) (the needle manufacturer was Elekta [Elekta, Veenendaal, the Netherlands], part number 083.920; diameter of 1.5 mm, and length of 140 mm).

All CT images were transferred to Onecentra 4.3 treatment planning software (Elekta Brachytherapy, Veenendaal, The Netherlands) to formulate the brachytherapy plan. Target volumes—including high-risk clinical target volume (HR-CTV), intermediate-risk clinical target volume (IR-CTV), and OARs (bladder, rectum, and sigmoid colon)—were depicted on CT images. The HR-CTV included the entire cervix and residual tumor during brachytherapy. In contrast, IR-CTV included all components of the HR-CTV and areas with tumor infiltration before external beam IMRT [12]. The dose curves were optimized repeatedly using manual/graphic optimization with Oncentra 4.3 treatment-planning software (Elekta Brachytherapy, Veenendaal, The Netherlands) to ensure that the prescribed doses for the curves were lower around the HR-CTV and OARs. Tumor target and OAR doses were calculated based on the equivalent dose delivered in 2 Gy fractions (EQD2). The doses were as follows: for HR-CTV D90, EQD2 ≥ 85 Gy; for bladder D2cm^3^, EQD2 ≤ 90 Gy; and for rectum and sigmoid D2cm^3^, EQD2 ≤ 75 Gy [13]. D90 referred to the dose at 90% of the HR-CTV, and D2cm^3^ referred to the dose received by 2 cm^3^ of the OAR.

### Deriving the sub-organs from the OAR

**Fig. 1:**
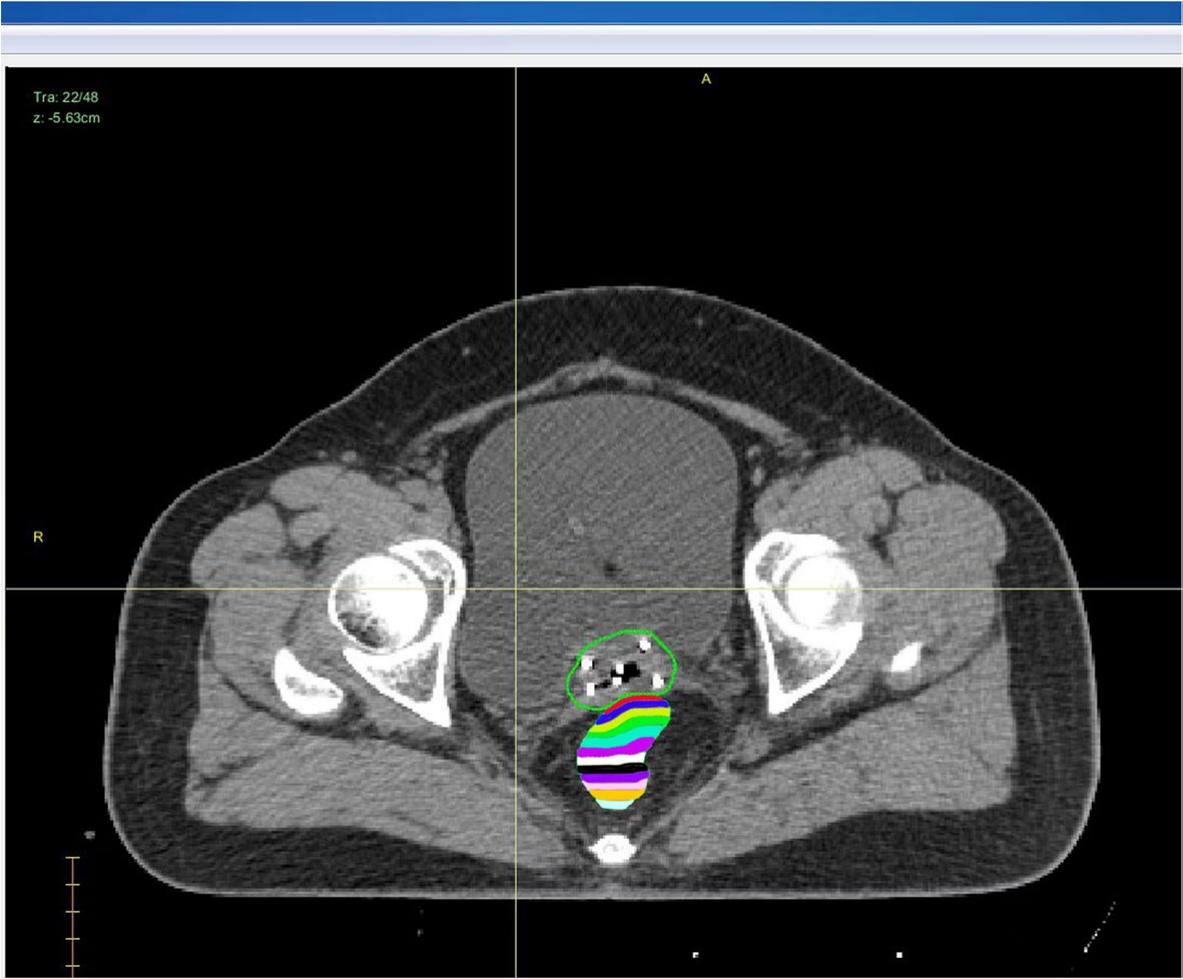
Sub-organ of the rectum The blue line indicates HR-CTV; the shadow area indicates the sub-organ

The HR-CTV was externally expanded to a plurality of rings (ring_1_–ring_n_) with a width of 0.3 cm. Ring_1_–ring_n_ and different OAR (bladder, rectum, and sigmoid colon) intersection regions (ring_1_–ring_n_ ∩ OAR) were used as independent sub-organs, with ring_n_ ∩ OAR defined as sub-organ-n (e.g., ring_1_ ∩ OAR was defined as sub-organ-1). The total number of sub-organs was kept under 15 (the intersecting regions for sub-organ-1 to sub-organ-5 of the rectum are presented in Fig. 1). The sub-organ volume was normalized for improved data analyses. The normalized sub-organ volume (V_nsub-organ_) was equal to the sub-organ volume divided by the OAR volume. In this study, the MATLAB application written by us was used to automatically generate the sub-organ and automatically read as V_nsub-organ_. One of the patients automatically generated a CT tomogram of the sub-organ for the rectum through a self-written MATLAB app program (Fig. 1).

### Dose prediction model based on the neural network method

The D2cm^3^/D90 of the OAR was used as the prediction target to eliminate the influence of different D90 of the HR-CTV. Correlations between D2cm^3^/D90 for each OAR and their V_nsub-organ_—as well as the HR-CTVs for the bladder, rectum, and sigmoid colon—were analyzed.

The neural network prediction model was established based on a correlation, and the predictive model was established using the MATLAB neural net-fitting application (R2017a, MathWorks, Inc., Natick, MA, USA). We selected the Levenberg–Marquardt (LM) algorithm to train 100 iterations through the MATLAB neural net-fitting application, and we chose the best result to establish the neural network prediction model of D2cm^3^/D90 (output) with V_nsub-organ_ and the HR-CTVs of the bladder, rectum, and sigmoid colon as the input variables. The network consisted of three layers: (1) the input layer (transmission rate), which receives input data to the network through a set of neurons; (2) the hidden layer, which runs a set of algorithms to compute the input data; and (3) the output layer linear transformation, which iteratively calculates the desired result through a set of linear output neurons. The output depends on the weighted sum of the input variables plus bias to ensure numerical stability [14]. The LM algorithm applied in the network was a back-propagation algorithm, which is a combination of two minimization algorithms and a gradient descent algorithm—i.e., the Gauss–Newton algorithm [15].

### Predictive accuracy evaluation

The neural network prediction model was evaluated using the regression R-value (Regression R values measure the correlation between outputs and targets. An R value of 1 indicates a close relationship, and 0 indicates a random relationship). In addition, the mean squared error (MSE) was the average squared difference between outputs and targets (lower values are better, and zero indicates no error). Model performance was also quantified as follows: ∆D2cm^3^/D90 =|D2cm^3^/D90(actual)-D2cm^3^/D90(predicted)| (mean and standard deviation).

### Statistical methods

We conducted Pearson’s correlation test to analyze the correlation between D2cm^3^/D90 of each OAR with HR-CTV volume, each OAR volume, and their V_nsub-organ_ using Statistical Product Service Solutions (SPSS) 19.0. Statistical differences between predicted and planned values of D2cm^3^/D90 of each OAR were compared using paired *t* tests. *P* < 0.05 was considered statistically significant.

## Results

The average number of needles inserted per patient was 3.7, with a maximum of eight and a minimum of two needles. The correlation analysis results of D2cm^3^/D90 for the bladder, rectum, and sigmoid colon with HR-CTV volume, bladder volume, rectal volume, sigmoid colon volume, and their respective sub-organ volumes are shown in Tables 1. and 2. As our results showed a significant correlation between D2cm^3^/D90 and related parameters, we then used the neural network-based method to establish the predictive model.

**Table 1:** Correlation coefficient between D2cm^3^/D90 of each OAR and the volumes of HR-CTV, bladder, rectum and sigmoid colon

D2cm^3^/D90	Variable				
	Volume of HR-CTV	Volume of bladder	Volume of rectum	Volume of sigmoid colon	Volume of small intestine
D2cm^3^ D90 (bladder)	0.539^a^	0.349^a^	\	\	\
D2cm^3^/D90 (rectum)	0.454^a^	0.195^a^	0.154^a^	\	\
D2cm^3^/D90 (sigmoid colon)	0.424^a^	0.189^a^	\	0.391^a^	\

Note: ^a^ significant correlation at 0.01 level (bilateral)

**Table 2:** Correlation coefficient between D2cm^3^/D90 and V_nsub-organ_ of each OAR

D2cm^3^/D90	V_nsub-organ_				
	V_nsub-organ1_	V_nsub-organ2_	V_nsub-organ3_	V_nsub-organ4_	V_nsub-organ5_
D2cm^3^/D90 (bladder)	0.391^a^	0.485^a^	0.476^a^	0.419^a^	0.334^a^
D2cm^3^/D90 (rectum)	0.220^a^	0.601^a^	0.743^a^	0.773^a^	0.753^a^
D2cm^3^/D90 (sigmoid colon)	0.286^a^	0.402^a^	0.487^a^	0.506^a^	0.536^a^

^a^Significant correlation at 0.01 level (bilateral)

**Fig. 2:**
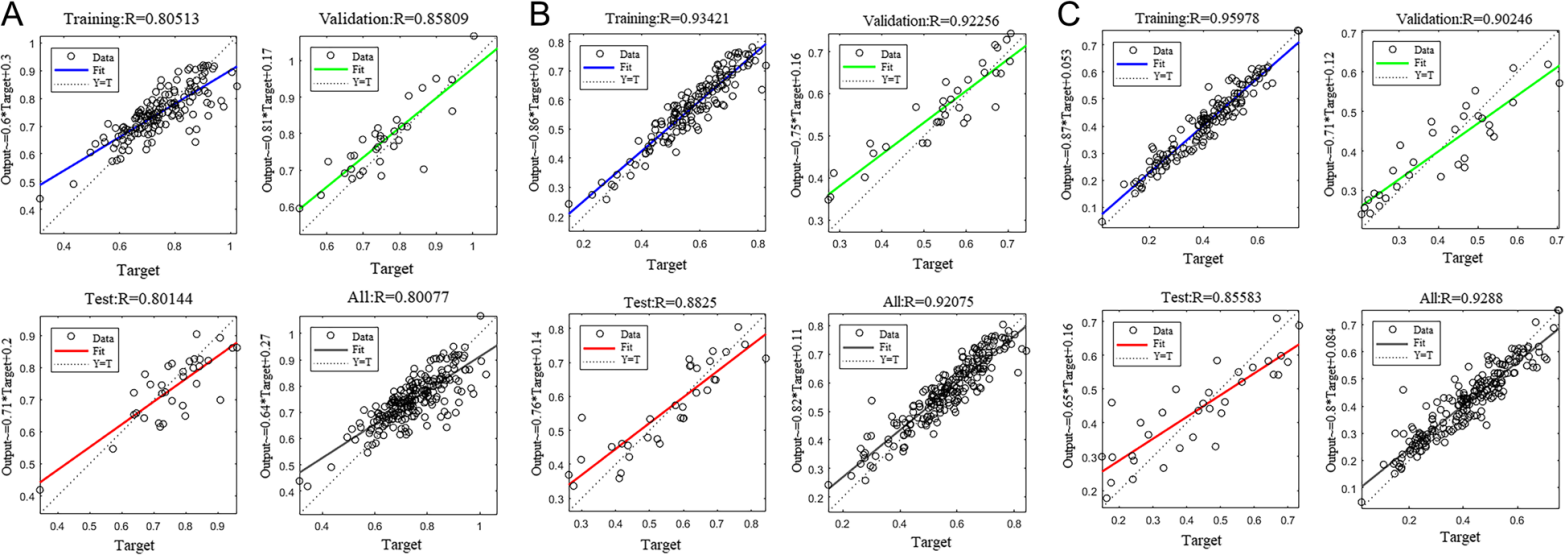
Regression diagram of Dn0–Dn50 for the predictive model: **A** bladder, **B** rectum, and **C** sigmoid colon

**Fig. 3:**
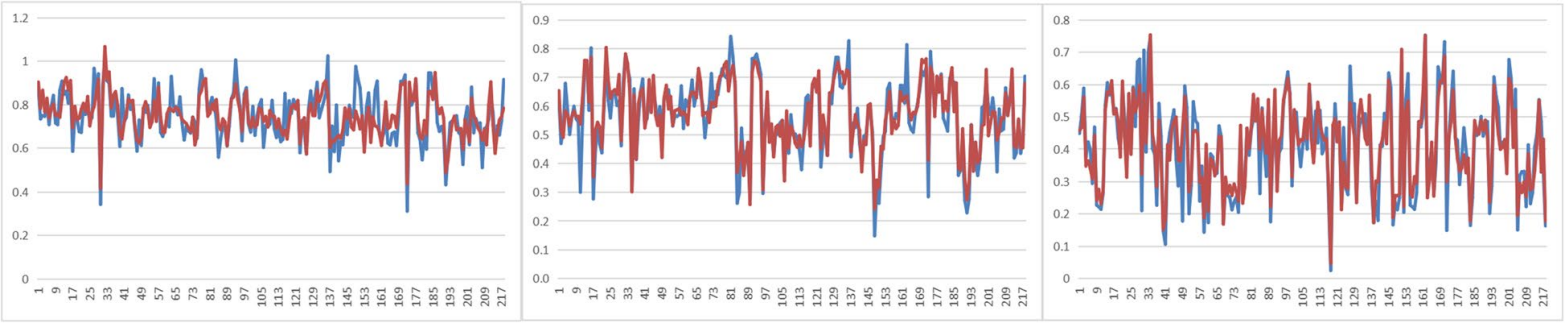
Predicted value and the actual planned value for all sets. Red indicates the predicted value of the neural network model; blue indicates the actual planned value: **A** bladder, **B** rectum, and **C** sigmoid colon

The R values for the bladder, rectum, and sigmoid colon in the training set for the predictive model were 0.80513, 0.93421, and 0.95978, respectively. The mean values for the ∆D2cm^3^/D90 for the bladder, rectum, and sigmoid colon in all sets were 0.052 ± 0.044, 0.040 ± 0.032, and 0.041 ± 0.037, respectively. The R values for the bladder, rectum, and sigmoid colon in the validation set for the predictive model were 0.85809, 0.92256, and 0.90246, respectively; in the testing set for the predictive model were 0.80144, 0.88250, and 0.85583, respectively; and in all sets for the predictive model were 0.80077, 0.92075, and 0.92880, respectively. We observed no statistical difference between the D2cm^3^/D90 predicted value for each OAR and the actual planned value in all sets (*P* > 0.05). The p-value in our paired *t* test results for D2cm^3^/D90 for the bladder, rectum, and sigmoid colon were 0.630, 0.185, and 0.638, respectively (*P* > 0.05). The MSEs for the bladder, rectum, and sigmoid colon in the training set for the predictive model were 4.779 × 10^−3^, 1.967 × 10^−3^, and 1.574 × 10^−3^, respectively. The regression diagram of the prediction model is shown in Fig. 2; the MSE for the predictive model is presented in Table 3; and the predicted and actual plots for all set are depicted in Fig. 3.

**Table 3:** MSE value of neural network prediction model for D2cm^3^/D90

OAR	Set			
	Training	Validation	Testing	all
bladder	4.779 × 10^–3^	3.499 × 10^–3^	5.543 × 10^–3^	4.702 × 10^–3^
rectum	1.967 × 10^–3^	2.965 × 10^–3^	5.028 × 10^–3^	2.576 × 10^–3^
sigmoid colon	1.574 × 10^–3^	3.934 × 10^–3^	8.815 × 10^–3^	3.027 × 10^–3^

## Discussion

With the continuous development of artificial intelligence (AI) in radiation oncology (RO) and interventional radiotherapy (IRT, brachytherapy), AI and automation in RO and IRT are able to successfully facilitate all the steps of treatment workflow. Compared to traditional approaches, AI exhibits potential benefits in reducing time-consuming repetitive tasks and improving treatment-plan quality assurance [16]. Implementing AI in IRT might also result in significant advantages for physicians, allowing them more time to interact with patients. Several recent studies have underlined the concept that AI can automatically adjust the weight parameters of treatment plans, assist in optimizing applicator location in treatment planning phases, and predict the optimal source position in targets—thus avoiding irradiation of OARs in brachytherapy [17–19]. Through machine learning analysis of pre- and post-plan seed configurations, effective algorithms have been developed to obtain sufficient target coverage and optimal OAR avoidance in brachytherapy [20, 21]. OAR dose prediction has developed into an exciting area in the application of AI in radiotherapy, and dose prediction has been widely applied to IMRT to reduce time-consuming repetitive tasks and assuring the quality of IMRT plans [22–24]. For OAR dose prediction in cervical cancer brachytherapy, Damato et al. [25] deployed a dataset of 20 patients to develop a simple mathematical model and predict bladder and rectal D2cm^3^ for gynecological interstitial brachytherapy, with the parameters they used for prediction being the overlapping volume of the organ at risk with the targeted area or a 1-cm expansion of the target area. In contrast to the work of Damato et al., we possessed a larger patient plan with additional parameter input for prediction. Yusufaly et al. [26] applied an approach closely related to that developed for IMRT to predict bladder, rectum, and sigmoid D2cm^3^ for tandem and ovoid treatments. Reijtenbagh et al. [27] used 3D U-NET CNN to perform voxel-based dose prediction on OARs, and Cortes et al. [28] used overlap volume histograms (OVHs) to evaluate dose prediction on OARs. In contrast to their work, our model only included inputs for the volume of sub-organs of the OAR and did not require voxel information to establish a predictive model; thus, even planners without programming experience can use the model based on open-source software (MATLAB, etc.).

The current method was successfully applied in previous studies to the dose prediction of IMRT for nasopharyngeal carcinoma and achieved favorable results [29]. In contrast to the work of machine learning methods [8–11], our model was easy to establish for radiotherapy planners, and it does not require complex modeling. With this study, we were the first to uncover a correlation between D2cm^3^/D90 for each OAR in brachytherapy using needle insertion and their sub-organs, and to establish a predictive model based on the neural network method. Any institution can enter the sub-organ volume for each OAR to predict OAR doses. Furthermore, we did not note any significant differences in model performance among training, validation, and test sets, indicating that the model was not biased towards patients in the training set and did not overfit.

We also analyzed ∆D2cm^3^/D90 to evaluate model performance, where the absolute value was used for evaluation; thus, this indicator accurately reflected model performance. ∆D2cm^3^/D90 for the bladder, rectum, and sigmoid colon in all sets were 0.052 ± 0.044, 0.040 ± 0.032, and 0.041 ± 0.037, respectively; with no statistical difference detected between the D2cm^3^/D90 predicted value for each OAR and the actual planned value. The MSE of the bladder, rectum, and sigmoid colon in the training set for the predictive model were 4.779 × 10^−3^, 1.967 × 10^−3^, and 1.574 × 10^−3^, respectively; and the R value for the predictive model was greater than 0.8, while in the rectum and sigmoid colon, the R value was greater than 0.9. Based on these results, we hypothesize that the predictive model is valid and stable.

If the radiotherapy planning system is used to divide each OAR into sub-organs and record their volumes, then the amount of data is enlarged and the time cost remains high, and this is not conducive to the promotion of the current research method. Therefore, in this study we independently wrote our own MATLAB application, with the compilation automatically generating the sub-organ for each OAR and automatically reading V_nsub-organ_. The entire process did not require manual participation, greatly improving overall efficiency.

Our research is presently limited to one institution; thus, our proposed method may reflect certain limitations. We expect that additional studies will further increase multicenter research in this area. As the number of patient plans increases, more plan data can be merged to ensure that we obtain a more accurate predictive model.

Despite the limitations to the current model, its predictive accuracy indicates that the model can still be used as a quality control tool for brachytherapy plans. In the actual application of brachytherapy plans, the threshold value of the difference between the predicted value and planned value can be set with respect to the quality control of the plan. If the difference between the two exceeds the threshold, then the plan should be further optimized so as to reduce the influence of subjective factors for planners and to carry out quality control and quality assurance for the individualized plan of brachytherapy. Open-source software was used in this study, and the required arguments were read directly from the planning system or from a written MATLAB application. For developing countries (particularly for newly established radiotherapy departments), the quality control tool for the brachytherapy plan can be established without using any other new software modules for radiotherapy planning.

We adopted a new and simpler dose prediction method to predict the critical OAR D2cm^3^ quality indicator for patients with cervical cancer receiving brachytherapy. To our knowledge, this study was the first to show a correlation between D2cm^3^/D90 for each OAR and its sub-organ in cervical cancer brachytherapy using needle insertion. Based on these conditions, we postulate that the OAR dose prediction model that we have established will greatly improve the quality control and automation of patient treatment plans. This method has also obtained a Chinese invention patent.

## Data Availability

All data generated and analyzed during this study are included in this published article.
